# The Development of Volumetric Quantitative Evaluation Software for Assessing Respiratory-Induced Target Motion

**DOI:** 10.7759/cureus.72978

**Published:** 2024-11-04

**Authors:** Hideharu Miura, Masao Tanooka, Soichiro Ishihara, Masahiro Kenjo, Minoru Nakao, Shuichi Ozawa, Masayuki Kagemoto

**Affiliations:** 1 Department of Radiation Oncology, Hiroshima High-Precision Radiotherapy Cancer Center, Hiroshima, JPN; 2 Department of Radiation Oncology, Hiroshima University, Institute of Biomedical &amp; Health Sciences, Hiroshima, JPN; 3 Department of Radiotherapy, Takarazuka City Hospital, Takarazuka, JPN

**Keywords:** deformable image registration, four-dimensional computed tomography, lung, respiratory induced organ motion, vector volume histogram

## Abstract

Purpose

We developed a volumetric quantitative evaluation software called vector volume histogram (VVH) to evaluate respiratory-induced organ motion using deformable image registration (DIR).

Methods

The B-spline-based DIR algorithm was used to compute the deformation vector field (DVF), which included the DVF_LR_ (left-right), DVF_AP_ (anterior-posterior), and DVF_CC_ (craniocaudal). The VVH software was written as a plug-in using Python, thus allowing anyone to easily modify the code. A shifted target within the moving phantom was used to evaluate the performance of the VVH software. The 2 cm diameter target was systematically shifted by 5, 10, 15, and 20 mm in the CC direction. To evaluate respiration-induced target motion, the VVH method was applied during the inhalation and exhalation phases of 4D CT scans in a patient with lung cancer. Length at 5% volume (L_5%_) and length at 50% volume (L_50%_​​​​​) were calculated to evaluate the target motion.

Results

In the phantom study, the VVH software accurately measured target displacements with L_5%_ and L_50%_ values of 5.4 mm and 4.8 mm, 10.4 mm and 9.8 mm, 14.9 mm, and 14.6 mm, and 19.9 mm and 19.6 mm for 5, 10, 15 and 20 mm displacements, respectively. For the lung cancer patient study, the VVH method showed target motion with L_5%_ and L_50%_ values of 1.9 mm and 1.8 mm in LR, 1.9 mm and 0.9 mm in AP, 18.8 mm and 15.8 mm in CC, and 18.9 mm and 15.8 mm in 3D vector. The centroid method measured respiratory tumor motion between the inhalation and exhalation phases as 0.5 mm, 0.7 mm, 13.5 mm, and 13.5 mm in the LR, AP, and CC directions and in the 3D vector.

Conclusions

The VVH software provided a volumetric quantitative assessment of respiratory-induced target motion and may provide strategic decisions for clinical use at the time of treatment planning.

## Introduction

Respiratory motion management in radiotherapy is critical to ensure precise and accurate delivery of radiation to the tumor while minimizing exposure to surrounding healthy tissues [1]. Respiratory motion management is recommended when the magnitude of the organ or tumor motion is greater than 5 mm. Various approaches to respiratory motion management in radiotherapy include breath holding, abdominal pressure, respiratory gating, and tumor tracking. The respiratory motion management method is based on the measured data of respiration-induced tumor motion during a computed tomography (CT) simulation session. Four-dimensional computed tomography (4D-CT) is the primary method used to evaluate the respiratory motion of a tumor in three dimensions (3D). Variations in the internal morphology and deformation over the free-breathing cycle can be observed with 4D-CT. Several researchers have studied lung tumor motion and determined that the lower lobe of the lung has a greater motion amplitude than the other lobes [2-4]. However, most data were measured at only a few points, namely the centroid and edge, and these measurements do not consider organ deformation.

Deformable image registration (DIR) has been used for dose accumulation, response assessment, organ delineation, and adaptive radiotherapy (ART) owing to its ability to create a new deformed image [5]. DIR is an important tool for estimating organ deformation and displacement. Previously, we proposed a quantitative volumetric method called the vector volume histogram (VVH) to evaluate respiratory-induced organ motion using DIR [6, 7]. VVH can evaluate the volumetric organ motion at the pixel level for each organ. In our previous study, the calculated deformation vector field (DVF) in the treatment planning system (TPS), which has a DIR function, was exported using a script. The DVF was then converted to digital imaging and communications in a medicine-radiation therapy (DICOM-RT) dose file format using in-house software. The converted DICOM-RT file was analyzed using a dose-volume histogram (DVH) function on the TPS. This process is highly complicated and makes use of the VVH method challenging.

The purpose of this study was to develop a volumetric quantitative evaluation software to assess respiratory-induced organ motion using DIR. The VVH method was applied to a series of moving phantoms to demonstrate the utility of this computational approach through graphical and indexed representations of the spatial deformation distribution. Additionally, the VVH method was applied to a patient with lung cancer for clinical use.

This article was previously presented as a session abstract at the 2024 ASTRO Annual Meeting, September 29 - October 2, 2024.

## Materials and methods

Vector volume histogram

The VVH software was implemented on the commercial DIR software iVAS (ITEM Corporation, Osaka, Japan) as a plug-in software using Python v3.6. The B-spline-based DIR algorithm implemented in the iVAS was used in this study, and its accuracy was validated in a previous study [8]. The VVH software required two image sets for the DIR. The first image set was the reference image, and the second was the registered image. DIR was used to deform the first images into the second images and calculate the DVF. The VVH is a calculation method similar to the standard dose array and DVH. A DVF is a three-dimensional array that defines the deformation of each pixel. The left-right (LR), anterior-posterior (AP), and cranio-caudal (CC) vectors were stored in separate arrays. The 3D DVF was calculated using the following expression: $\tiny\sqrt{DVF_{LR}^{2}+DVF_{AP}^{2}+DVF_{CC}^{2}}$ . Each DVF value is calculated as an absolute value.

The volumetric index for the motion of each organ was calculated by evaluating the number of DVF values in the organ, such as the dosimetric index for plan evaluation (e.g., D95%, D98%, and D2%). Respiratory-induced tumor motion was evaluated using the following defined motion indices: minimum length (L_min_), maximum length (L_max_), length at 95% volume (L_95%_), length at 50% volume (L_50%_), and length at 5% volume (L_5%_). In the VVH software, the user can change the result of the direction by selecting a combo box. Other features include a calculation index, changing the range of the x- and y-axes, a tracking bar, and export to a CSV file.

Phantom study

A displaced target within a phantom was used to evaluate the performance and accuracy of the VVH system under conditions that mimicked respiratory-induced tumor motion in a clinical setting. A CIRS phantom (Dynamic Thorax Phantom Model 008A; Computerized Imaging Reference Systems, Norfolk, MA, USA) was used to create different target positions. The CIRS phantom consisted of a static thorax phantom and a lung-equivalent rod containing a spherical water-equivalent target. The target can be moved in the LR, AP, and CC directions by rotating and translating the rod. A CT image without a moving target was defined as the reference CT image. The 2 cm diameter target was systematically shifted by 5, 10, 15, and 20 mm in the CC direction to simulate varying tumor amplitudes. The phantom was scanned using an Optima CT scanner (GE Medical Systems, Waukesha, WI, USA), and images were acquired using a slice thickness of 1.25 mm.

Patient study

A lung cancer patient who underwent SBRT was selected to verify the performance of the VVH system. The patient underwent 4D CT scans under free-breathing conditions and was immobilized in the supine position with arms extended above the head. The respiratory motion of the patient's abdomen was recorded using a Varian real-time position management (RPM) respiratory gating system (Varian Medical Systems, Palo Alto, CA, USA). The CT images had a slice thickness of 2.5 mm with a gantry rotation time of 1.0 s. The reconstructed 4D CT images were sorted using Advantage 4D software (Varian Medical Systems, Palo Alto, CA, USA) into 10 respiratory phases according to the corresponding phase of the respiratory cycle, with 0% corresponding to end-inspiration and 50% corresponding to end-exhalation. The inhalation and exhalation phases of the 4D CT images were analyzed using the VVH software. The gross tumor volume (GTV) was delineated by a radiation oncologist. Inhalation and exhalation CT images were used as the primary and secondary image datasets, respectively. The results of the VVH method were compared with those obtained using the centroid method.

## Results

**Figure 1:**
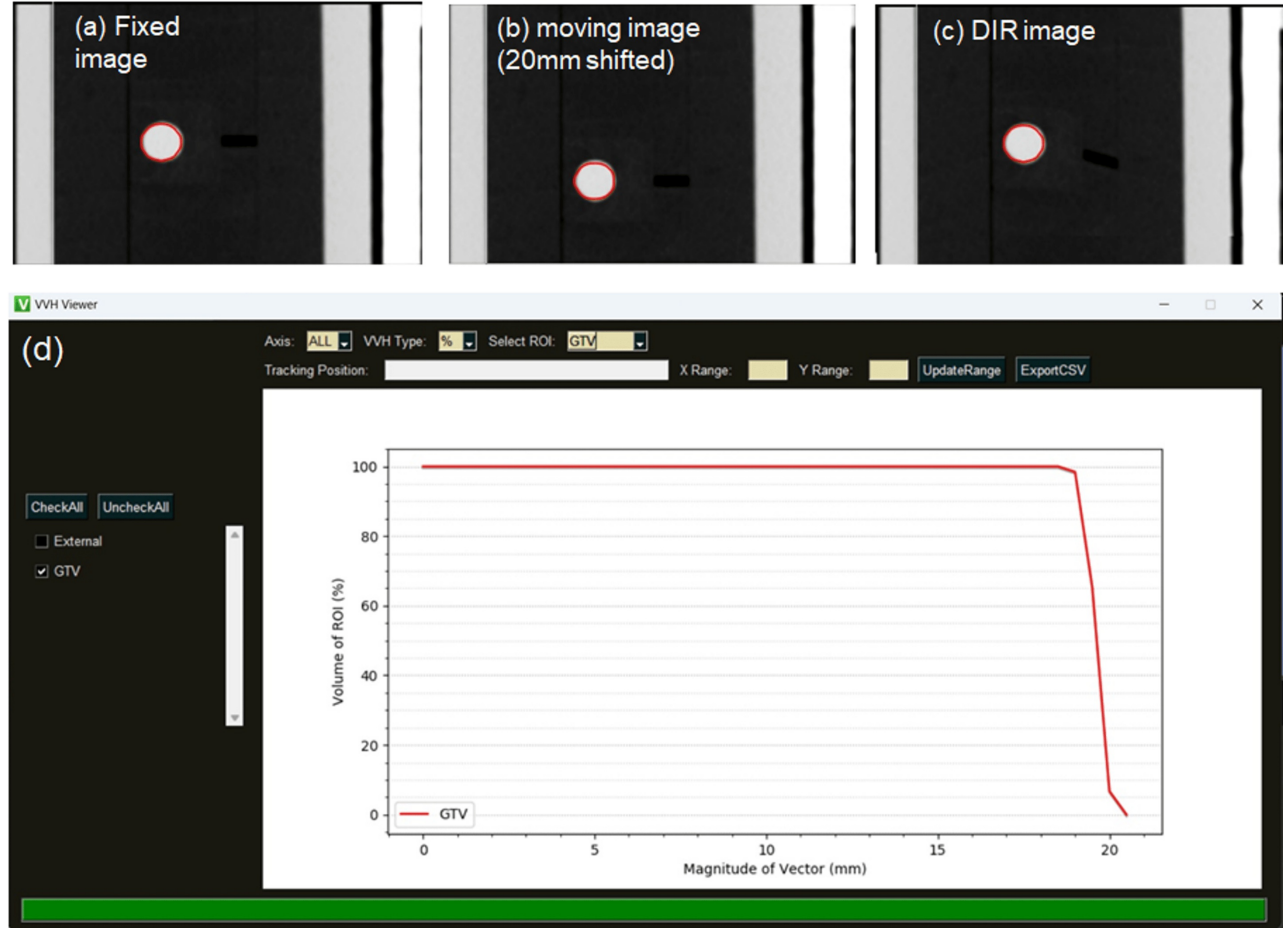
Screenshot of vector volume histogram (a) fixed image, (b) moving image, (c) deformable image registration (DIR) image, and (d) screenshot of the vector volume histogram (VVH) software, which can calculate the volumetric dose-volume histogram based on contouring.

Figure 1A-C shows the fixed CT, moving (20 mm offset), and DIR images. The DIR image was visually consistent with the fixed CT image. A screenshot of our VVH software for a 20 mm offset target is shown in Figure 1D.

**Figure 2:**
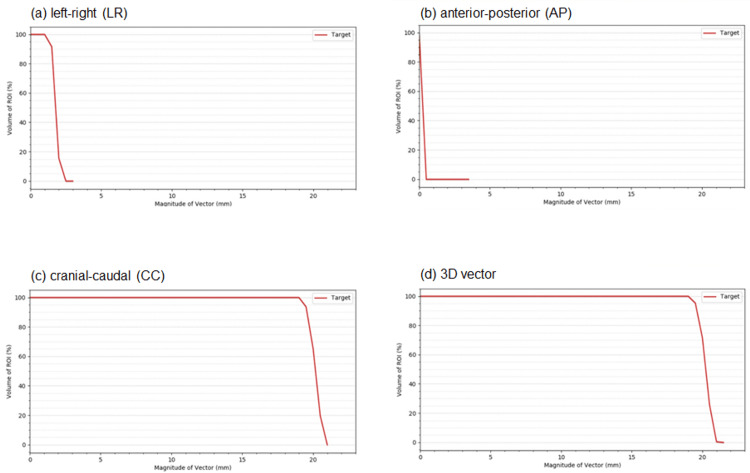
Vector volume histogram for phantom study Vector volume histograms (VVH) of a) left-right (LR), b) anterior-posterior (AP), c) cranial-caudal (CC) directions, and d) 3D vector, respectively, 20 mm target displacement.

Figure 2 shows the VVHs in the LR, AP, and CC directions and the 3D vector for a 20 mm target displacement.

**Figure 3:**
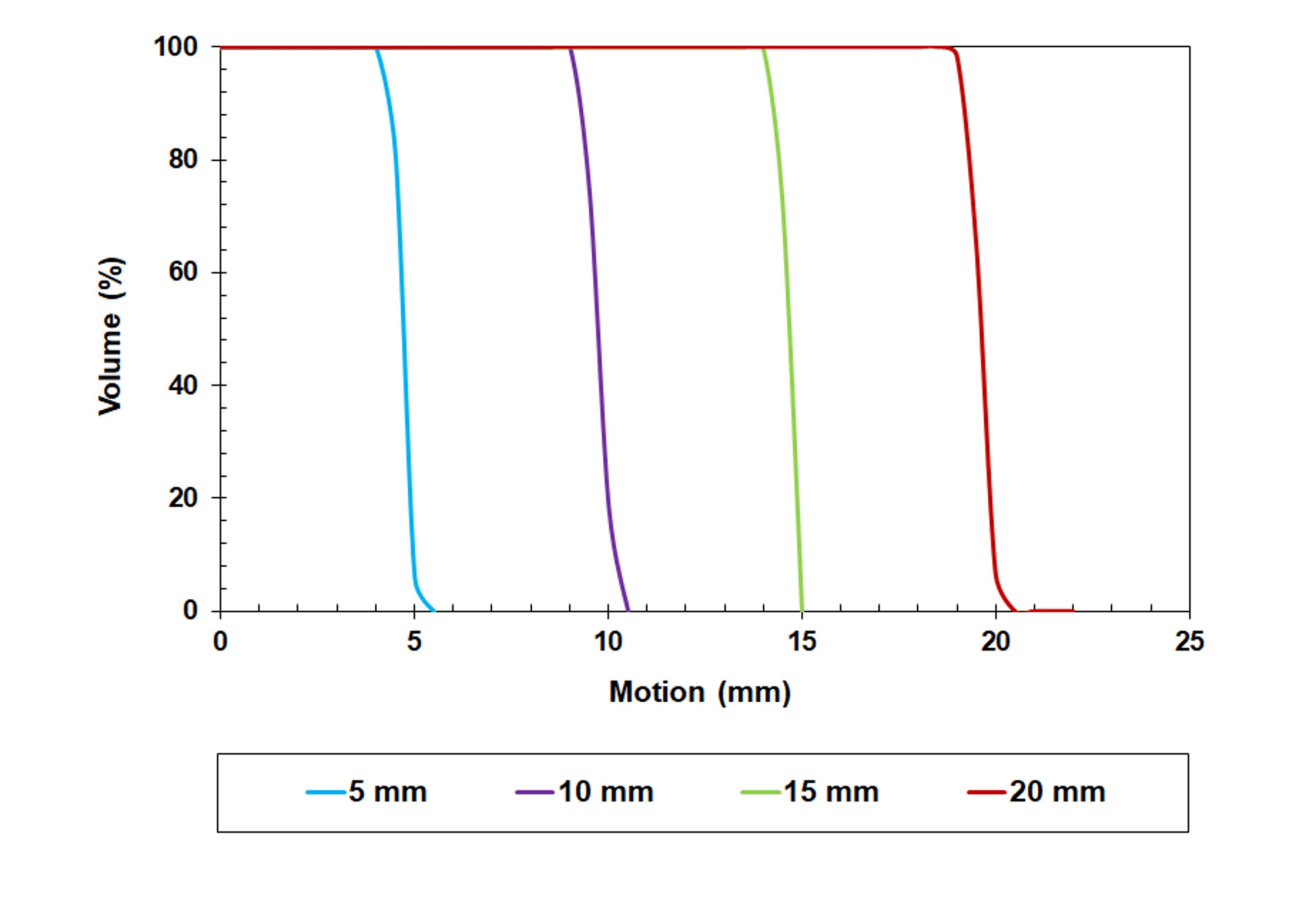
Vector volume histogram of 5, 10, 15, and 20 mm movement Vector volume histograms (VVH) for a target displacement of 5, 10, 15, and 20 mm in the cranial-caudal (CC) directions.

Figure 3 shows the VVHs in the CC direction for target displacements of 5, 10, 15, and 20 mm. The results of the VVH indices for the 3D vector for the target displacements of 5, 10, 15, and 20 mm are summarized in Table 1.

**Table 1:** Vector volume histogram index evaluation for moving target Lmin = minimum amount of movement within the organ, Lx% = X% of the organ moved by at least this length, Lmax = maximum amount of movement within the organ

Index	5 mm	10 mm	15 mm	20 mm
L_min_	3.9 mm	8.9 mm	14.0 mm	18.9 mm
L_95%_	4.2 mm	9.2 mm	14.1 mm	19.1 mm
L_50%_	4.8 mm	9.8 mm	14.6 mm	19.6 mm
L_5%_	5.4 mm	10.4 mm	14.9 mm	19.9 mm
L_max_	5.5 mm	11.5 mm	15.0 mm	20.1 mm

**Figure 4:**
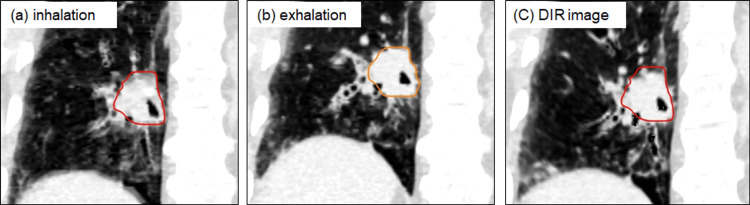
Clinical case (a) Inhalation and (b) exhalation CT images and (c) the deformable image registration (DIR) results using the B-spline.

Figure 4 shows the inhalation and exhalation CT images and the DIR results obtained using the B-spline. In the centroid method, the respiratory tumor motion between the inhalation and exhalation phases for this patient was 0.5, 0.7, 13.5, and 13.5 mm in the LR, AP, and CC directions and the 3D vector, respectively.

**Figure 5:**
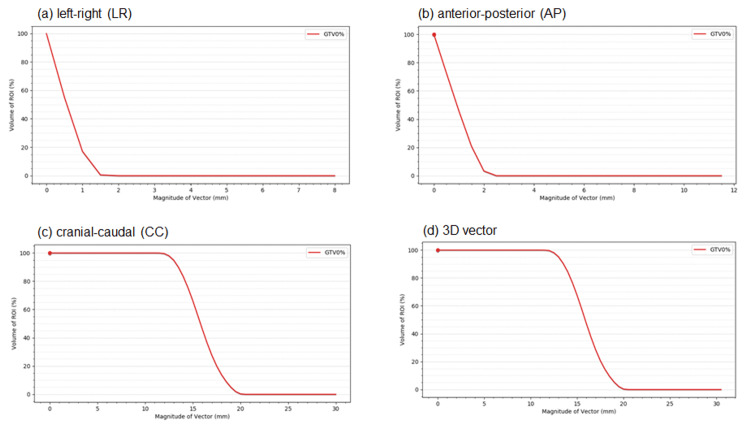
Vector volume histogram for clinical case Vector volume histograms (VVH) of (a) left-right (LR), (b) anterior-posterior (AP), (c) cranial-caudal (CC) directions, and (d) 3D vector of a lung cancer patient.

Figure 5 shows the VVHs in the LR, AP, and CC directions, and the 3D vector for a patient with lung cancer. The VVH indices in the LR, AP, and CC directions and in the 3D vector for the patient with lung cancer are summarized in Table 2.

**Table 2:** Vector volume histogram index evaluation for patient case Lmin = minimum amount of movement within the organ, Lx% = X% of the organ moved at least this length, Lmax = maximum amount of movement within the organ, LR = left-right, AP = anterior-posterior, CC = cranio-caudal

Index	LR	AP	CC	3D vector
L_min_	0.1 mm	0 mm	11.9 mm	12.0 mm
L_95%_	0.3 mm	0.1 mm	13.0 mm	13.1 mm
L_50%_	1.8 mm	0.9 mm	15.8 mm	15.8 mm
L_5%_	1.9 mm	1.9 mm	18.8 mm	18.9 mm
L_max_	2.5 mm	2.4 mm	19.8 mm	19.9 mm

## Discussion

We developed a method to evaluate volumetric quantitative evaluation software for assessing respiratory organ motion and demonstrated the utility of VVH for phantom studies. The target motion was accurately deformed and measured volumetrically using the VVH method. The mean difference in the target motion between the VVH results (L_95%_, L_50%_, and L_5%_) and the known target motion was less than 1 mm, which was considered within acceptable limits for clinical use, although VVH results depended on the volumetric index used. Because it depends on the size of the DIR calculation grid, Lmin and Lmax are appropriate for determining tumor motion. Intensity-based DIR algorithms are significantly affected by the local feature content in the images, such as contrast, noise, and anatomical locations [9, 10]. After confirming the visual assessment of DIR, the VVH results should be carefully analyzed.

The results of the centroid method were influenced by delineation, and the results of the VVH may not be fully comparable. The relative ratio index can be used to evaluate the volumetric motion measurement. The centroid method is suitable for round tumors; however, it is not suitable for volumes with very complicated shapes, where the delineation is uncertain. Compared to the centroid method, VVH is cumbersome to use in external software and should be more user-friendly. VVH can be used to detect the presence and provide a quantitative index of large motions and deformations within the structures of interest, although no spatial location information is provided. Our current system adds a development mode based on a commercial system that is user-friendly, has no additional training time, and is easy to understand, using a concept similar to DVH. Our VVH program code can also be modified by a user using Python.

In previous studies, Kirby et al. calculated the cumulative error in the overall spatial accuracy of 11 DIR algorithms [11]. Shi et al. used the DVF error to evaluate the accuracy of DIR algorithms in three widely used commercial systems [12]. Wang et al. proposed a DIR error analysis tool called the deformation error histogram (DEH) that calculates deformation errors per anatomical structure [13]. Mayyas et al. generated DVF histograms by calculating the DVF for each voxel of the prostate and SVs and applied them to cone-beam computed tomography (CBCT) images [14]. Their in-house program is similar to our VVH method. Our ultimate goal is to promote the quantitative volumetric method; therefore, we created a plug-in for the commercial DIR software.

In this study, the assessment of tumor motion amplitude was performed by measuring the difference in tumor position between peak exhalation and peak inspiration using 4D CT imaging and did not consider hysteresis effects that may occur over multiple respiratory cycles. Lung tumors cause volumetric deformation of size and shape throughout 4D CT scans [15, 16]. All phases of the respiratory tumor motion on 4D CT can be evaluated using the proposed VVH method. We applied only the VVH method to CT-CT images. However, the VVH method with DIR can evaluate the interfractional organ motion using CBCT. A limitation of this study is that the VVH software was applied to only one patient. In a future study, we will further apply the VVH software to more clinical cases to determine respiratory management strategy decisions at the time of treatment planning. The optimal timing of decision-making in ART is challenging. This VVH software could serve as a clinical decision-support tool for objective decision-making regarding the optimal timing of adaptive radiotherapy. Spatial tumor motion and deformation relationships can be correlated with clinical outcomes.

In recent years, technological advances in the medical field have been rapid and continue to evolve [17]. One of the most revolutionary breakthroughs has been the introduction of the Internet of Things (IoT) concept into medical practice. Currently, radiotherapy institutions typically rely on dedicated software installed on-premises. The future development of cloud-based software could provide several benefits to users in terms of accessibility, data integration, and cost-effectiveness.

## Conclusions

The VVH software developed in this study demonstrated a volumetric quantitative assessment of respiration-induced organ motion for radiotherapy planning. This volumetric approach, implemented as a user-friendly plug-in for commercial DIR software, provides a more comprehensive assessment of target motion compared to traditional centroid-based methods. The phantom study demonstrated the accuracy of the software in measuring target displacements, with mean differences of less than 1 mm compared to known target motion. In the clinical case study, the VVH method provided detailed volumetric motion data with L_5%_ values of 1.9 mm, 1.9 mm, and 18.8 mm in the LR, AP, and CC directions, respectively. This level of detail allows for more informed decision-making regarding respiratory motion management strategies during treatment planning. In addition, the VVH approach shows promise as a potential tool for determining optimal timing in adaptive radiotherapy. By better understanding organ motion and deformation, this software has the potential to improve the precision of radiation delivery.
